# Insights on the Synthesis of N-Heterocycles Containing Macrocycles and Their Complexion and Biological Properties

**DOI:** 10.3390/molecules27072123

**Published:** 2022-03-25

**Authors:** Fouad Malek, Tarik Harit, Mounir Cherfi, Bonglee Kim

**Affiliations:** 1 Laboratory of Applied Chemistry and Environment—ECOMP, Faculty of Sciences, Mohammed First University, Bd Mohamed VI, Oujda 60000, Morocco; cherfi_mounir1718@ump.ac.ma; 2 Department of Pathology, College of Korean Medicine, Kyung Hee University, Seoul 02447, Korea

**Keywords:** azole, biological activity, coordination properties, macrocycle

## Abstract

Macrocyclic chemistry has been extensively developed over the past several decades. In fact, the architecture of new macrocyclic models has undergone exponential growth to offer molecules with specific properties. In this context, an attempt is made in this study to provide an overview of some synthetic methods allowing the elaboration of N-heterocycles containing macrocycles (imidazole, triazole, tetrazole, and pyrazole), as well as their applications in the complexation of metal cations or as pharmacological agents.

## 1. Introduction

The design and synthesis of new macrocyclic architectures as large receptor compounds have received considerable attention in recent years owing to their encapsulating properties toward several guests [1]. This makes them highly important in multiple potential applications in molecular recognition, transport, biological models. or selective catalysis [1,2,3]. The importance of macrocyclic chemistry is also associated with a large number of natural complex macrocycles, including chlorophyll, haemoglobin, and vitamin B12, in which the receptors are porphyrin rings, and guests are magnesium, iron, or cobalt ions, respectively. In addition, nonactin and valinomycin are another class of natural oxygen macrocycles known as natural antibiotics that are obtained from *Streptomyces* species [4,5]. In 1967, Pedersen et al. [6] described the crown ethers as the first macrocycles exhibiting selective complexation properties toward the alkali and alkaline earth metal cations. This pioneer finding was followed by the elaboration of cryptands and spherands of Lehn and Cram, respectively [7,8] who were awarded the Nobel Prize in 1987 [9]. Thereafter, the design and synthesis variability of such macrocyclic systems exponentially increased by varying the cavity size and nature, and the number of the donor atoms as well as the attached lateral arms through changing the ratio and substituents of starting materials [10]. Such structural and electronic modifications have been extended to the introduction of N-heterocycle rings such pyrazole, imidazole, or pyrazine into the motif cycle to increase the number of *sp*^2^-hybridised N-donor atoms [11] and, consequently, to improve the ability of the corresponding macrocycles to complex both hard and soft cations such as alkali and transition metal cations of different oxidation states [12]. In this context, several studies in the literature have provided reviews that summarise the synthesis of some N-heterocyclic macrocycles. In 2008, McGinley and Fleming [13] reviewed some studies on the macrocycles containing tetrazole functional groups. Recently, Yang et al. highlighted recent advances in the synthesis and structure of N-heterocyclic carbenes based on macrocycles and their applications [14]. As a continuation to these pioneering studies, the focus of the present review is to give the reader deep insights into the synthesis of some kind of macrocyclic molecules reported in recent decades, as well as some pertinent details related to their complexion and biological properties.

## 2. Synthesis of Imidazolic Macrocycles

**Figure 1:**
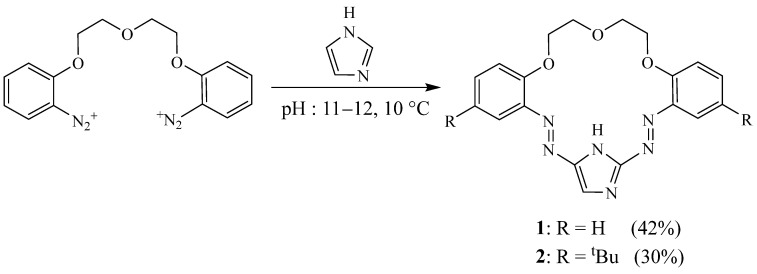
Final step of the synthesis of macrocycles **1** and **2** (Reprinted with permission from ref. [15]. Copyright 2003 Elsevier).

In 2003, Wagner-Wysiecka et al. [15] reported the synthesis of two imidazole-based macrocyclic chromogenic derivatives **1** and **2** by coupling imidazole with the bis-diazonium salts (Figure 1) under basic medium (pH = 11–12) and high dilution conditions, respectively). These compounds were obtained in medium yields (42% and 30% for **1** and **2**). This study revealed that these imidazole-based macrocycle ligands coordinate preferentially alkali and alkaline earth cations and the ion-selective membrane electrodes doped with such imidazole derivatives are sodium-selective electrodes.

**Figure 2:**
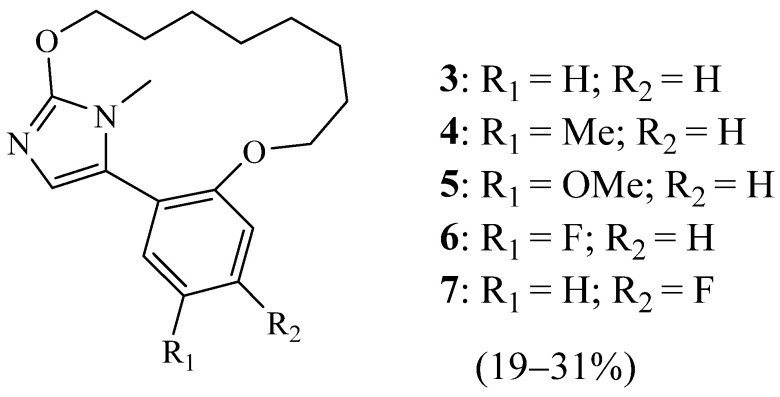
Structures of macrocycles reported by Nshimyumukiza et al. [16] (Reprinted with permission from ref. [16]. Copyright 2010 Elsevier).

A few years later (2010), Nshimyumukiza et al. [16] described a new family of compounds based on the 5-aryl-1*H*-imidazole motif (Figure 2), for which the chemical synthesis involves a three-step sequence: aromatic nucleophilic substitution (SNAr), Suzuki coupling, and ring-closing metathesis (RCM) reaction [17]. This method allowed the preparation of a variety of novel macrocyclic substrates **3**–**7** in good overall yields. Biological evaluation of synthesised imidazole-containing macrocycles revealed that they display good binding activity toward the A3 adenosine (h) receptor, dopamine D1 (h) receptor, chloride channel (GABA-gated), and choline transporter (h) CHT1.

**Figure 3:**
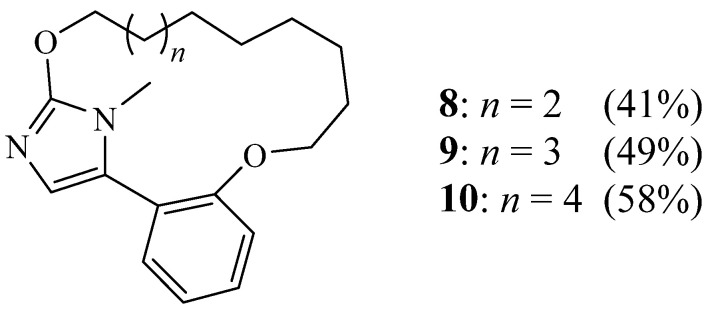
Structures of macrocycles **8**–**10** (Reprinted with permission from ref. [18]. Copyright 2011 John Wiley and Sons).

Van Den Berge et al. also used principally the same method to elaborate other 5-aryl-1*H*-imidazole-containing macrocycles **8**–**10** by varying the size cavity (Figure 3) [18]. They found that the cyclisation step yield increased with an increase in the chain length. Biological evaluation of the two optically pure enantiomers of one of these molecules enabled them to investigate the influence of chirality on biological activities.

**Figure 4:**
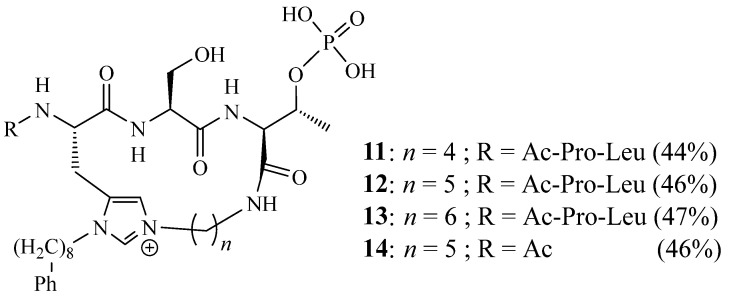
Structures of macrocycles **11**–**14** (Reprinted with permission from ref. [19]. Copyright 2018 Elsevier).

Hymel et al. [19] reported the synthesis of tripeptide ligands with decreased molecular weight **11**–**14** (Figure 4). This was conducted by *C*-terminal macrocyclisation, employing N(π),N(τ)-bis-alkylated residues as ring junctions and showing improved target selectivity for the polo-box domain of polo-like kinase 1 (Plk1 PBD) versus the PBDs of Plk2 and Plk3.

**Figure 5:**
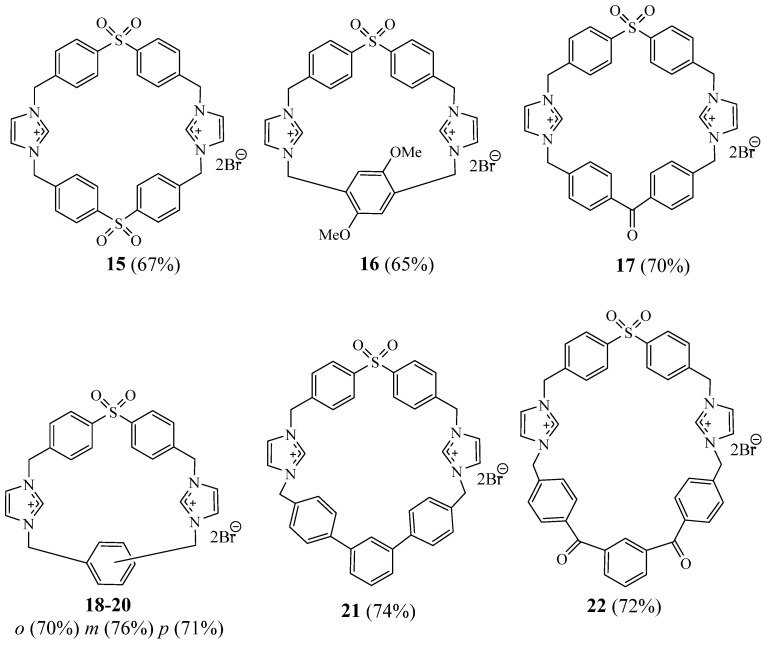
Structures of macrocycles **15**–**22** (Reprinted with permission from ref. [20]. Copyright 2011 Elsevier).

Rajakumar et al. [20] reported the synthesis of some novel imidazole-based dicationic sulphonophanes **15**–**22** incorporating various spacer units by capping a precyclophane with a suitable dibromide (Figure 5). They found that all the obtained sulphonophanes exhibit good antibacterial and antifungal activity against five bacterial strains *Bacillus subtilis*, *Staphylococcus aureus*, *Vibrio cholera*, *Escherichia coli*, *Proteus vulgaris*, and the human pathogenic fungus *Candida albicans*.

**Figure 6:**
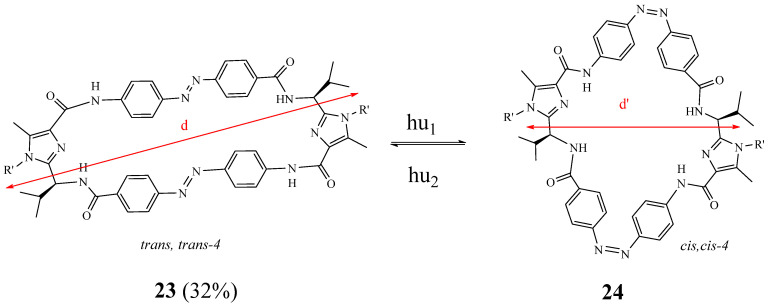
Structures of conformational macrocycles **23** and **24** (Reprinted with permission from ref. [21]. Copyright 2018 John Wiley and Sons).

Mehrparvar et al. [21] reported the synthesis and structural investigation of a platform consisting of two imidazole amino acids, which are connected through two azobenzene units **23** and **24** (Figure 6). This platform can be switched by light from the elongated *trans*, *trans*-isomer **23** to the compact *cis*, *cis* isomer **24**, and back.

**Figure 7:**
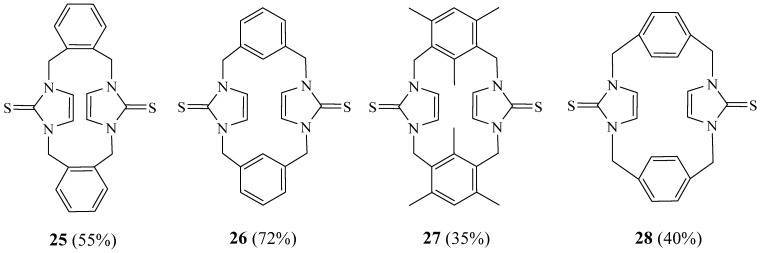
Structures of cyclophanes **25**–**28** (Reprinted with permission from ref. [22]. Copyright 2018 Elsevier).

Mageed et al. [22] described the synthesis of new cyclophanes **25**–**28** (Figure 7) containing two imidazole-2-thione moieties linked by two xylylene groups by the reaction of imidazolium-linked cyclophanes with sulphur in the presence of K_2_CO_3_ and using methanol as solvent. Structures of the new cyclophanes were confirmed and investigated by NMR spectroscopy, as well as by X-ray diffraction studies.

**Figure 8:**
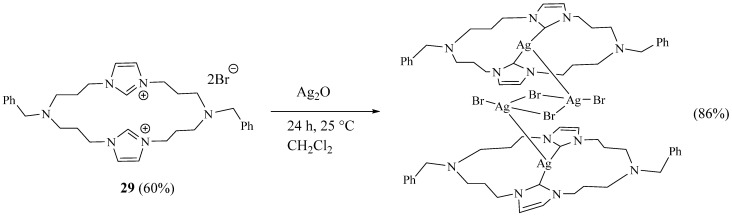
Imidazolic macrocycle **29** and its silver complex (Reprinted with permission from ref. [23]. Copyright 2019 Royal Society of Chemistry).

Recently, Thapa and Kilyanek [23] communicated the synthesis of a new macrocycle **29** consisting of a 20-membered ring containing two imidazolium salt functionalities in five steps. The last one is the condensation of a mixture containing N-benzylbis(3-imidazolpropyl) amine and N-benzylbis(3-bromopropyl)amine in high dilution to prevent possible oligomerisation side reactions, and the macrocycle was obtained in 60% yield. The reaction of this macrocyclic salt with silver oxide afforded bis-macrocyclic silver (I) complexes (Figure 8).

**Figure 9:**
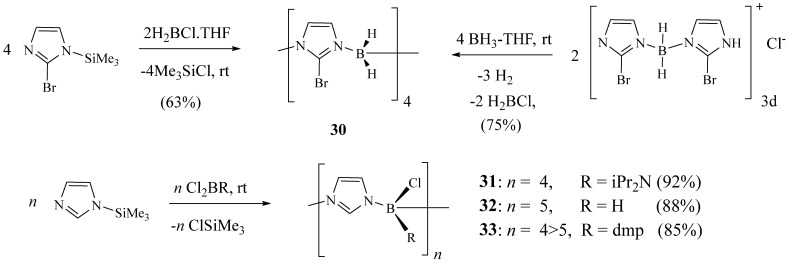
Synthetic pathway of macrocyclic imidazolylboranes **30**–**33** (Reprinted with permission from ref. [24]. Copyright 2003 Elsevier).

Weiss et al. [24] synthesised a new family of macrocyclic imidazolylboranes, by reacting 1-trimethysilylimidazoles and haloboranes XB(R_1_)_2_ through the boron/silicon exchange using 2-bromoimidazole **30**–**33** (Figure 9). The resulting macrocycles had the zwitterionic character and contain imidazolyl rings linked through their nitrogen atoms by BH_2_. They also employed a new synthetic strategy to prepare these macrocyclic imidazolylboranes, including the preparation and cyclisation of bis(imidazolyl)boronium chlorides.

**Figure 10:**
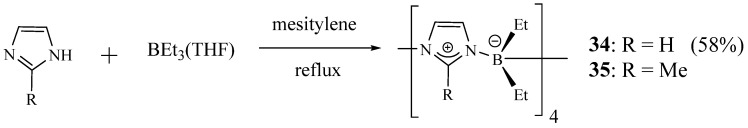
Tetrameric boron–imidazole macrocycles **34** and **35** (Reprinted with permission from ref. [25]. Copyright 2012 Elsevier).

Iwanek et al. [25] presented a very simple and efficient synthesis of tetrameric boron–imidazole macrocycles **34** and **35** involving the reaction of imidazole or 2-methylimidazole and triethylborane (Figure 10). The synthesis of these macrocycles was performed in two steps. First, an equimolar amount of triethylborane in tetrahydrofuran was added to imidazole or 2-methylimidazole, followed by refluxing for about 1 h. Next, the tetrahydrofuran was evaporated, mesitylene was added, and the mixture was refluxed at mesitylene boiling temperature for several hours.

**Figure 11:**
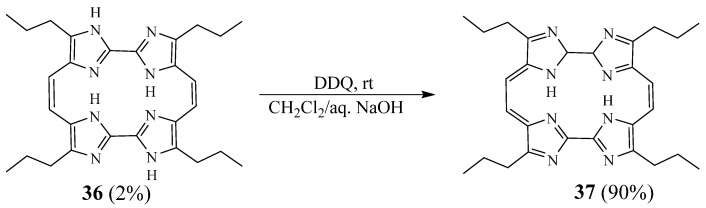
Synthetic route of macrocycle **37** (Reprinted with permission from ref. [26]. Copyright 2003 John Wiley and Sons).

Sargent et al. [26] described the synthesis, spectroscopic properties, and computational analysis of an imidazole-based analogue of porphycene—namely, ‘imidacene’ **37**. The reductive coupling of a diformyl-substituted 2,2′-biimidazole using low-valent titanium gives the intermediate macrocycle **36**. This step was followed by treatment with 2,3-dichloro-5,6-dicyano-1,4-benzoquinone (DDQ) (Figure 11). This macrocycle was found to undergo rapid decomposition, even in the absence of light and air. Through high-level theoretical calculations, they explained this instability by the presence of a delocalised 18 π–electron pathway in both imidacene and porphycene that provides less aromatic stabilisation energy.

**Figure 12:**
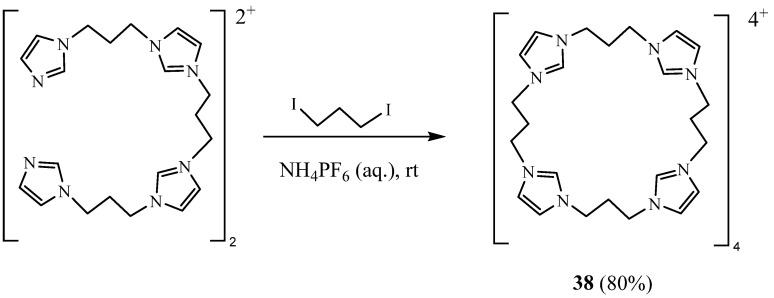
Synthesis of tetra-imidazolic macrocycle salt **38** (Reprinted with permission from ref. [27]. Copyright 2005 Royal Society of Chemistry).

Other new (tetrakis)imidazolium macrocyclic receptor system **38** was synthesised by Wong et al. [27]. This product was obtained using stepwise alkylation reactions of bis(imidazolium) precursor compound (Figure 12). They used the ^1^H NMR titration to study the binding properties of the resulting macrocycle toward halogen and benzoate anions in competitive conditions using acetonitrile–water (9:1) as solvent. Unfortunately, it was not possible for them to determine the stability constant values.

## 3. Synthesis of Tetrazolic Macrocycles

**Figure 13:**
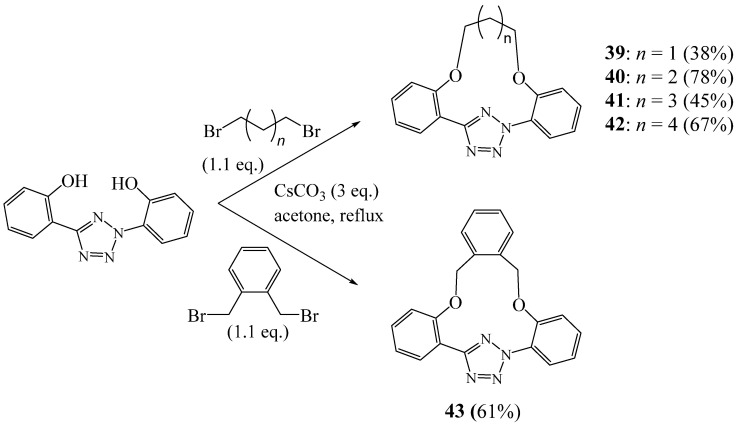
Synthesis of montetrazolic macrocycles **39**–**43** (Reprinted with permission from ref. [28]. Copyright 2010 John Wiley and Sons).

Yu et al. [28] presented the synthesis of a series of novel macrocyclic structures **39**–**43** incorporating one tetrazole ring by reacting dibromoalkanes with a tetrazole derivative in the presence of alkali metal bases (Figure 13). Through a systematic study, they provided evidence of the effect of the radius alkali cation on the yield of the synthesised macrocycles. They suggested that these macrocyclic tetrazoles should offer a new class of photoactivatable tetrazole reagents for the bioorthogonal tetrazole–alkene cycloaddition reaction in living systems.

**Figure 14:**
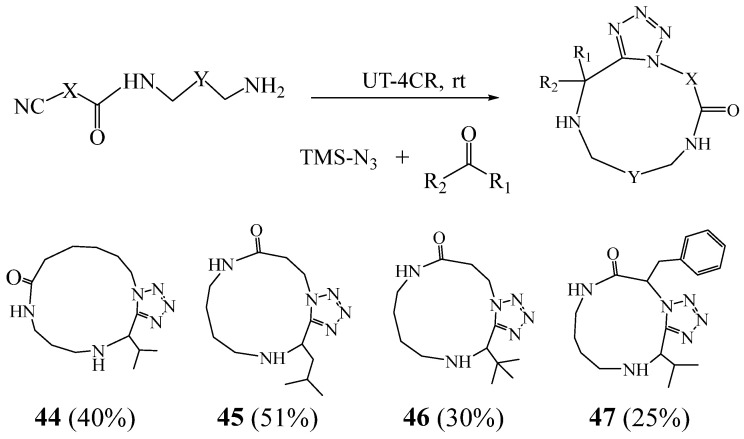
Monetetrazole containing macrocycles **44**–**47**.

Abdelraheem et al. [29] revealed the synthesis of another family of monotetrazole-containing macrocycles **44**–**47**, in two steps through accessible starting materials (Figure 14). The first step comprises a chemoselective amidation of amino acid-derived isocyanocarboxylic acid esters with unprotected symmetrical diamines to afford diverse α-isocyano-ω-amines. In the second step, the α-isocyano-ω-amines undergo an Ugi tetrazole reaction to close the macrocycle. This strategy allowed these authors short access to 11–19-membered macrocycles in which substituents could be independently varied at three different positions.

**Figure 15:**
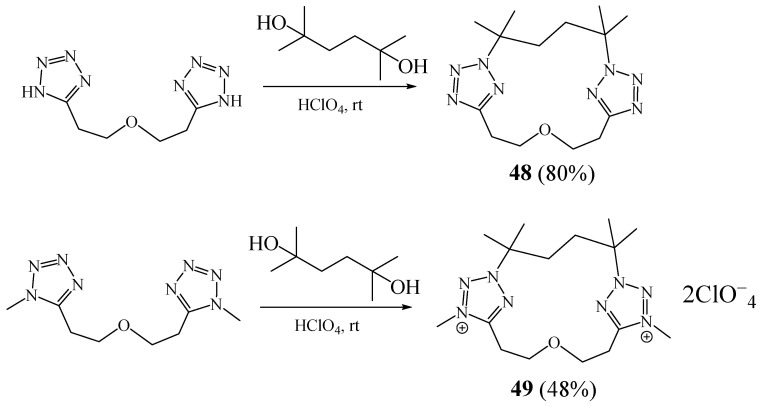
Elaboration of macrocycles **48** and **49** (Reprinted with permission from ref. [30]. Copyright 2012 Elsevier).

Voitekhovich et al. [30] described the synthesis of two new 15-membered macrocycles **48**, **49** with tetrazol-2,5-diyl moieties units linked by 3-oxapentane-1,5-diyl and 2,5-dimethylhexane-2,5-diyl bridges (Figure 15). Their synthesis involved condensation of 1,5-bis(tetrazol-5-yl)-3-oxapentane or 1,5-bis(1-methyltetrazol-5-yl)-3-oxapentane with 2,5-dimethylhexane-2,5-diol in 65% aqueous perchloric acid. Structures of these obtained macrocyclic compounds were confirmed by single-crystal X-ray analysis.

**Figure 16:**
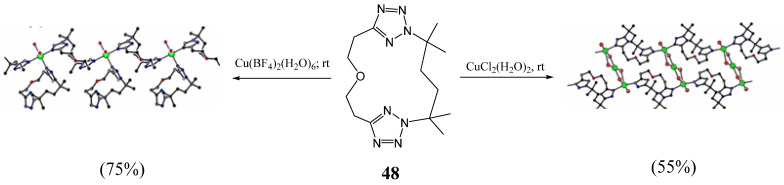
Copper complexes based on tetrazolic macrocycle **48** (Reprinted with permission from ref. [31]. Copyright 2017 American Chemical Societ).

The macrocycle **48** reacts with copper(II) chloride or copper(II) tetrafluoroborate hexahydrate to give complexes [Cu_3_Cl_6_**48**] and [Cu**48**(H_2_O)_2_](BF_4_)2(H_2_O) [31]. According to single-crystal X-ray analysis, both complexes were found to be coordination polymers (Figure 16).

**Figure 17:**
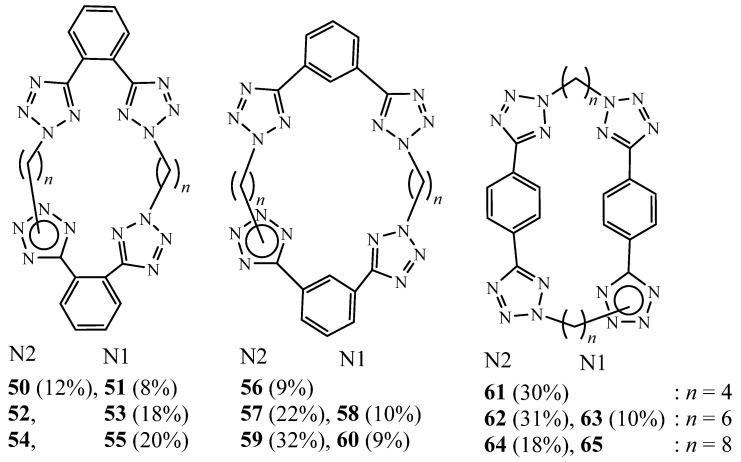
Structures of tetra-tetrazolic macrocycles **50**–**65** (Reprinted with permission from ref. [32]. Copyright 2007 Elsevier).

In 2007, Bond et al. described the syntheses of tetra-tetrazole macrocycles, containing two bis-tetrazole units **50**–**65** linked by a variety of alkyl chain lengths from 4–8 carbons by reacting one equivalent of 1,n-bis(tetrazol-5-yl)benzene and one equivalent of 1,2-, 1,3- or 1,4-[bis(2-(n-bromoalkyl)-tetrazol-5-yl)]benzene in dimethylformamide under nitrogen atmosphere and in the presence of potassium carbonate (Figure 17) [32]. The crystal structures of three of these derivatives were also reported. It was found that the macrocycle conformation is influenced by the length of the alkyl chain linker, the relative orientation of the tetrazole rings on the benzene ring, and by intermolecular interactions.

**Figure 18:**
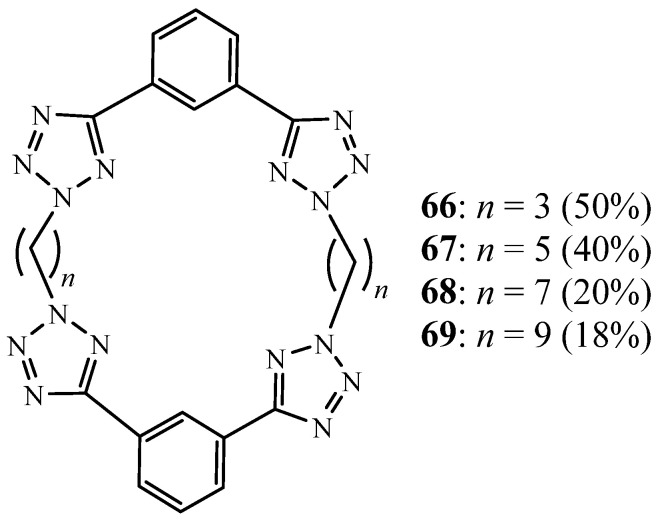
Tetrazolic macrocycles **66**–**69** (Reprinted with permission from ref. [33]. Copyright 2009 Elsevier).

Two years later, they revealed the syntheses of other tetra-tetrazole macrocycles **66**–**69**, containing two 1,3-bis(tetrazole)benzene units linked by a variety of *n*-alkyl chain lengths with an odd number of carbon atoms (*n* = 3, 5, 7, or 9 carbon atoms) (Figure 18) [33]. Tetra-tetrazole macrocycle (*n* = 7) contains an unexpected ‘host–guest’ interaction through the binding of a chloroform solvent molecule. The resulting deviation of the macrocycle from planarity results from a combination of the ‘host–guest’ interaction and strong intermolecular interactions between adjacent tetrazole and phenylene rings.

**Figure 19:**
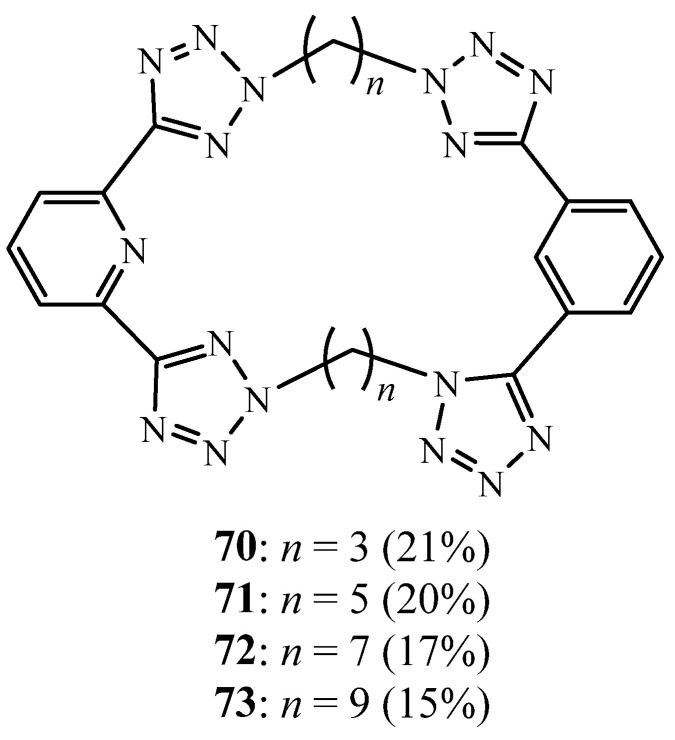
Tetrazolic macrocycle bearing pyridine moiety **70**–**73** (Reprinted with permission from ref. [34]. Copyright 2011 Elsevier).

The same group also substituted the phenyl by the pyridine ring to develop two new series of tetra-tetrazole macrocycles containing two 2,6-bis(tetrazole)pyridine units, linked by a variety of *n*-alkyl chain lengths **70**–**73** (Figure 19) [34]. The crystal structure of one of such tetra-tetrazole macrocycles was also structurally characterised and revealed a bowl-shaped conformation.

Teng et al. [35] studied theoretically complexes resulting from a tetra-tetrazolic macrocycle with some organic contaminants using density functional theory (DFT). They found that this tetra-tetrazole shows good binding affinity towards these molecules, and the stabilities of the formed complexes are affected by the number and effectiveness of the hydrogen bonds.

## 4. Synthesis of 1,2,4 Triazolic Macrocycles

**Figure 20:**
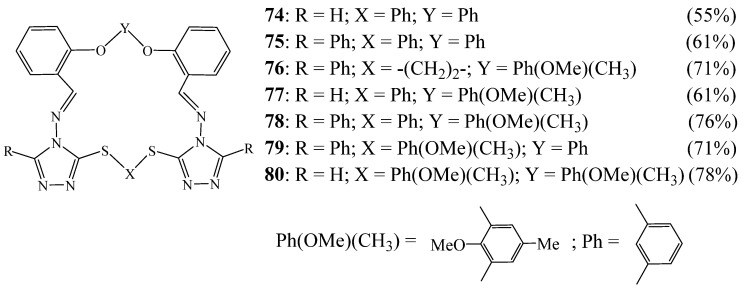
Structures of 1,2,4 Bitriazolic macrocycle **74**–**80** (Reprinted with permission from ref. [36]. Copyright 2007 John Wiley and Sons).

In 2007, Elwahy et al. [36] conveyed an elegant route for the synthesis of a series of novel macrocyclic Schiff bases containing two triazole rings **74**–**80** in good yields (Figure 20). They were obtained by heating bis-amines with the corresponding bis-aldehydes in refluxing acetic acid under high dilution conditions. Attempts to synthesise macrocyclic Schiff bases containing pyridine and two triazole rings were also described.

Brandt et al. [37] reported the synthesis of two lead (II) complexes of some sodium tri-macrocyclic Schiff salts bearing two 1,2,4 triazole moieties. This was performed by condensing a diketone with 1,3-diaminopropane or 1,4-diaminobutane through a 2+2 cyclisation in the presence of Pb(ClO_4_)_2_·3H_2_O. Transmetallation with nickel(II) ions yields a novel, structurally characterised, dinickel(II) macrocyclic complex.

**Figure 21:**
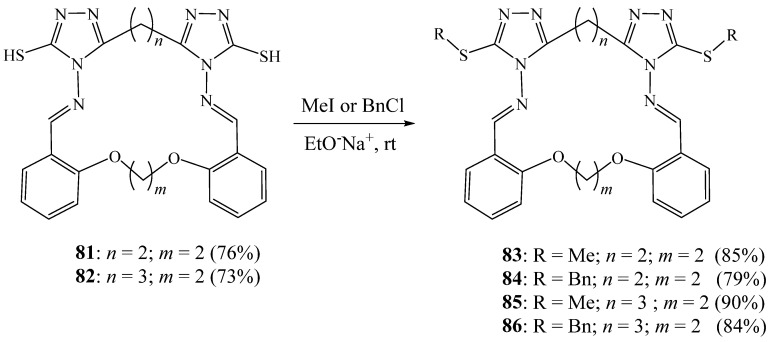
Alkylation of triazolic macrocycles **81** and **82** (Reprinted with permission from ref. [38]. Copyright 2009 © Georg Thieme Verlag KG).

Foroughifar et al. [38] prepared two new aza-crown macrocycles **81** and **82**, bearing two 1,2,4 triazolic rings by reacting of 1,2-, 1,3-, and 1,4-bis(4-amino-5-mercapto-4*H*-1,2,4-triazol-3-yl)alkanes with bisaldehydes in acetic acid under reflux. They were obtained in 70-76% yield. The reaction of these aza-crown macrocycles with iodomethane and benzyl chloride gave exclusively the target lariat macrocycles **83**–**86**, also in good yields (Figure 21).

**Figure 22:**
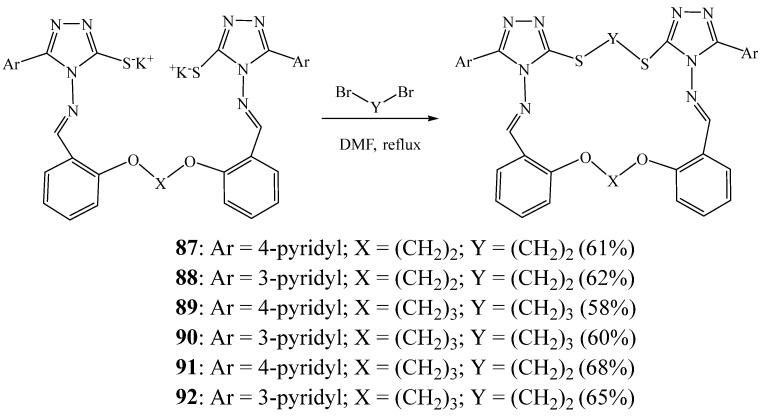
Cyclisation reaction to obtain macrocycles **87**–**92** (Reprinted with permission from ref. [39]. Copyright 2009 Elsevier).

They also reported a simple and efficient method for the preparation of azathia crown macrocycles **87**–**92** containing two triazole subunits [39]. First, a series of new 1,2/1,3-bis[*o*-(*N*-methylidenamino-5-aryl-3-thiol-4*H*-1,2,4-triazole-4-yl)phenoxy]alkane derivatives were prepared by condensation of 4-amino-5-(aroyl)-4*H*-1,2,4-triazole-3-thiols or 2-amino-5-mercapto-1,3,4-thiadiazole with bis-aldehydes. Then, the reaction of the obtained compounds with dibromoalkanes allowed obtaining the desired macrocycles (Figure 22). This method does not require high dilution techniques and provides the expected azathia macrocycles in good yields, ranging from 55% to 68%.

Avaji et al. [40] conveyed the synthesis of a series of tetra-triazolic macrocycles by condensing an equimolar ethanol mixture of 2,6-diformyl-4-methylphenol and bis-(4-amino-5-mercapto-1,2,4-triazol-3-yl)alkanes, and in the presence of a few drops of concentrated HCl. The macrocycles were obtained in good yields (60–65%). Due to the fact that they were found insoluble in common organic solvents, the corresponding La(III) and Th(IV) complexes were synthesised by template condensation of 2,6-diformyl-4-methylphenol, bis-(4-amino-5-mercapto-1,2,4-triazol-3-yl)alkanes and La(NO_3_)_3_ 6H_2_O/Th(NO_3_)_4_ 5H_2_O in 2:2:1 molar ratio in ethanol. The antimicrobial activities of macrocycles and their metal complexes were evaluated. Some compounds showed promising results.

Patil et al. [41] used procedures described by Avaji et al. [40] to elaborate four 1,2,4 tetra-triazolic macrocycles through a [2+2] cyclisation of Ortho-phthalaldehyde and bis-(4-amino-5-mercapto-1,2,4-triazole-3-yl)alkanes, as well as their corresponding Co^2+^, Ni^2+^ and Cu^2+^ complexes. The biological results demonstrated that all the Schiff bases possess antimicrobial activity, and their metal(II) complexes showed more promising activities than the Schiff bases. The interaction of copper(II) complexes with DNA was investigated by utilising gel electrophoresis. It was found that all copper(II) complexes cleave DNA efficiently.

**Figure 23:**
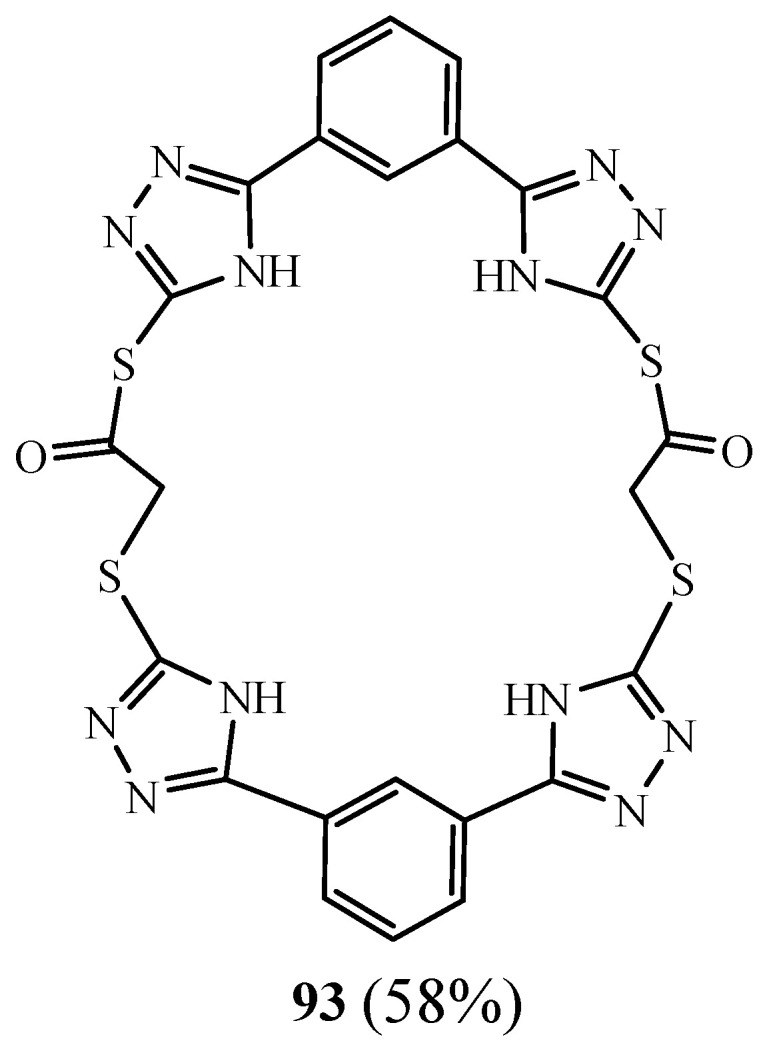
Structure of tetra-triazolic macrocycle **93** (Reprinted with permission from ref. [42]. Copyright 2012 John Wiley and Sons).

Kumar et al. [42] presented the synthesis of a new 1,2,4 triazolic macrocycle entitled *S*,*S*′-[benzene-1,3-diylbis(4*H*-1,2,4-triazole-5,3-diyl)]bis([(5-benzene-1,3-diyl-4*H*-1,2,4-triazol-3-yl)sulfanyl]ethanethioate) **93** (Figure 23) from isophthalic dihydrazide through a multistep reaction sequence. The desired compound and all intermediates were obtained in good yields (58–68%). Their antibacterial potency was evaluated against four different bacterial strains and was found comparable with that of the standard drug ciprofloxacin. The synthesised compounds were further studied for their possible in vitro antioxidant effects by DPPH scavenging, total antioxidant capacity, total reductive capacity, and H_2_O_2_ scavenging activity. It also possessed a good antioxidant activity when compared with the standards.

Chu Zheng et al. [43] sought to synthesise a new macrocyclic ligand with four 1,2,4 triazole subunits by reacting bis(5-amino-1*H*-1,2,4-triazole) and dichloromethane without metal ions but did not succeed. Nevertheless, they were able to elaborate on the corresponding metal complexes (Fe^2+^, Co^2+^, and Ni^2+^) in methanol as solvent. The fluorescence quenching spectra and UV–Vis spectra were used to study the interaction of complexes with bovine serum albumin (BSA).

## 5. Synthesis of 1,2,3 Triazolic Macrocycles

**Figure 24:**
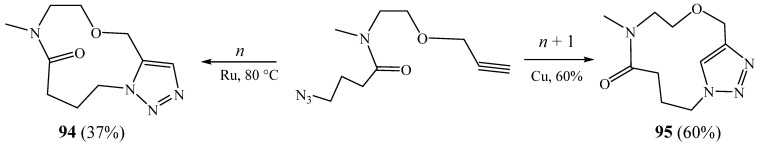
Effect of the catalyst nature on cyclisation (Reprinted with permission from ref. [44]. Copyright 2009 American Chemical Societ).

Kelly et al. [44] reported the synthesis of some mono 1,2,3 triazolic macrocycles through a regioselective intramolecular Huisgen cycloaddition, carried out on various azido alkyne substrates. Using catalyst control, a common intermediate was converted to two structurally unique macrocycles with either a 1,5- or a 1,4-triazole resulting in an *n* (**94**) or *n* + 1 (**95**) ring size by using Ru and Cu as a catalyst, respectively (Figure 24). 

**Figure 25:**
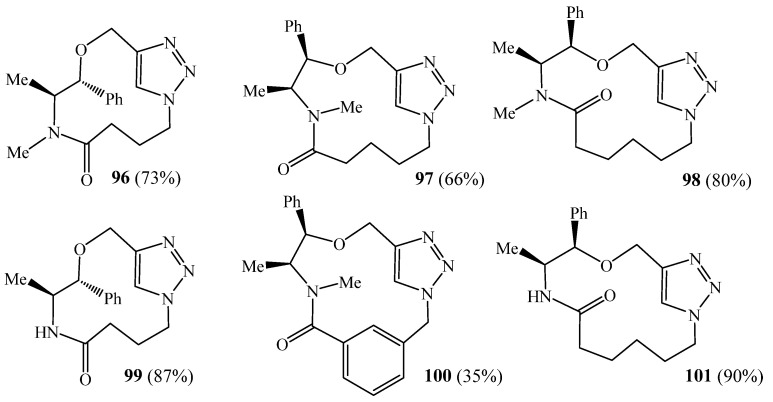
Structures of monotriazolic macrocycles **96**–**101** (Reprinted with permission from ref. [45]. Copyright 2010 John Wiley and Sons).

Bogdan et al. [45] generated a series of triazolic macrocycles **96**–**101** (Figure 25), with drug-like functionality and properties by simple and efficient copper-catalysed azide–acetylene cycloaddition reaction. These macrocycles were obtained in a 5 min reaction without resorting to the high-dilution conditions typical of macrocyclisation reactions, as well as in up to 90% yield.

**Figure 26:**
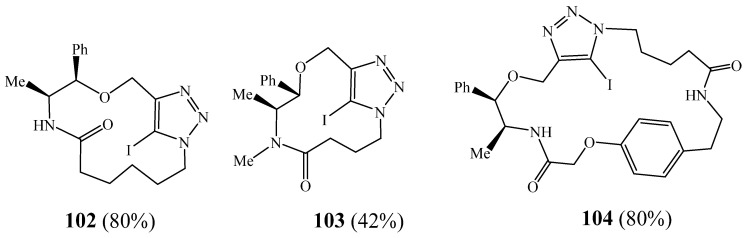
Structures of tetra-triazolic macrocycles **102**–**104** (Reprinted with permission from ref. [46]. Copyright 2011 American Chemical Societ).

The same team reported a new macrocyclisation strategy to synthesise 5-iodo-1,2,3-triazole-containing macrocycles **102**–**104** (Figure 26) [46]. The macrocycles were generated using a simple and efficient copper-catalysed cycloaddition in flow and under environmentally friendly conditions. This methodology also permits the facile, regioselective synthesis of 1,4,5 trisubstituted-1,2,3-triazole-containing macrocycles using palladium-catalysed cross-coupling reactions.

**Figure 27:**
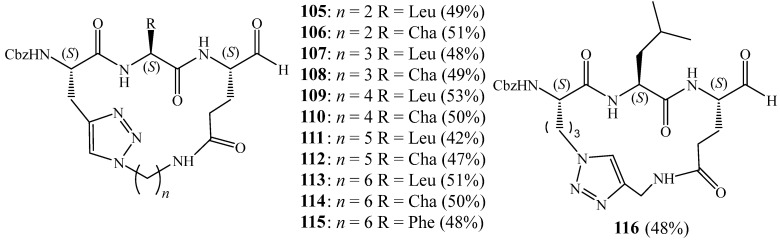
Structures of monatriazolic macrocycles **105**–**116** as inhibitors of norovirus 3CL protease (Reprinted with permission from ref. [47]. Copyright 2016 Elsevier).

Sessler et al. [47] also utilised the copper(I)-catalysed cycloaddition to synthesise cell-permeable 1,2,3 triazole-based macrocycles **105**–**116** with peptidyl backbone (Figure 27). The structure of these macrocycles was confirmed by NMR spectroscopy and high-resolution mass spectrometry (HRMS). They found that the obtained macrocycles could act as inhibitors of norovirus 3CL protease.

**Figure 28:**
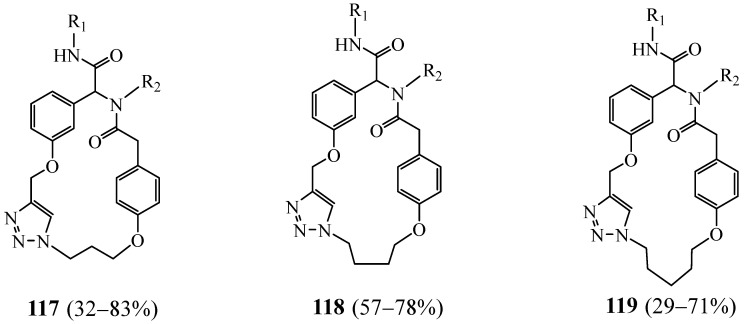
General structures of monatriazolic macrocycles **117**–**119** (Reprinted with permission from ref. [48]. Copyright 2018 John Wiley and Sons).

Hernández-Vázquez et al. [48] presented a multicomponent and rapid protocol for the synthesis of structurally diverse bis(aryl ether) macrocycles bearing one triazolic ring. This method allowed the synthesis of a family of 27 analogues with 20-(**117**), 21-(**118**), and 22-(**119**) membered rings (Figure 28). Some of the compounds displayed interesting cytotoxicity against cancer (PC-3) and breast (MCF-7) cell lines, especially those bearing an aliphatic or a trifluoromethyl substituent on the N-phenyl moiety (R_2_) (IC50 < 13 μM).

**Figure 29:**
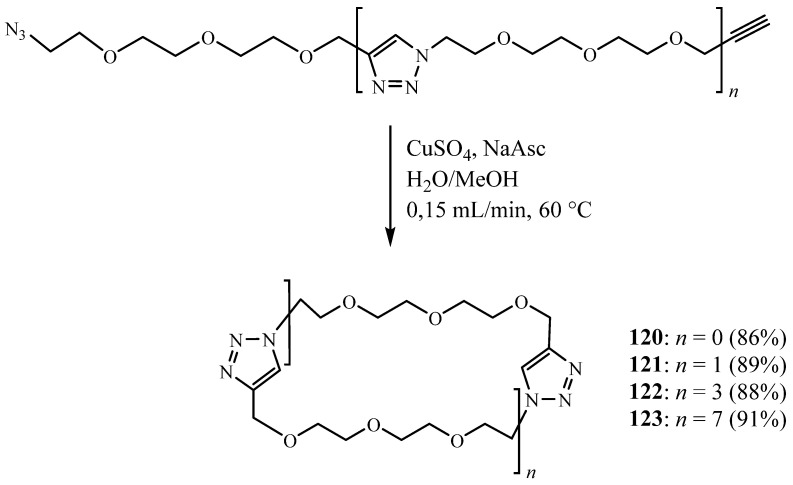
Structures of bitriazolic macrocycles **120**–**123** with different size cavities (Reprinted with permission from ref. [49]. Copyright 2009 John Wiley and Sons).

In their paper published in 2009, Sandra Binauld et al. [49] reported a subsequent CuAAC intramolecular cyclisation, performed under pseudo-high-dilution conditions, providing a series of novel macrocycles **120**–**123** with different ring sizes (Figure 29). This pathway was shown to be a facile, high-yielding process and can be accurately controlled.

**Figure 30:**
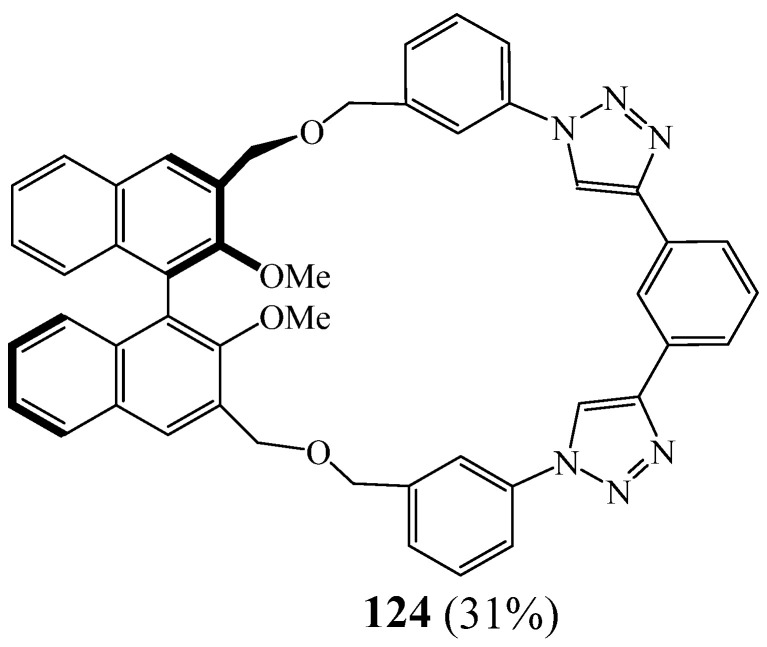
Structures of bitriazolic macrocycles **124** (Reprinted with permission from ref. [50]. Copyright 2012 Elsevier).

Caricato et al. [50] described the synthesis of bi-1,2,3 triazolic macrocycle **124** (Figure 30) through CuAAC ‘click’ reactions in the cyclisation step using toluene as solvent. Their methodology consists of fixing 1,2,3-triazole moieties within the macrocyclic backbone, which are able to directionally coordinate anions through CH⋯X^−^ hydrogen bonds.

Anandhan et al. [51] reported the synthesis of two triazole-based macrocyclic amides through click chemistry. They showed good anti-inflammatory activity even at low concentrations (50 µg/mL) when compared with that of the reference drug prednisolone.

**Figure 31:**
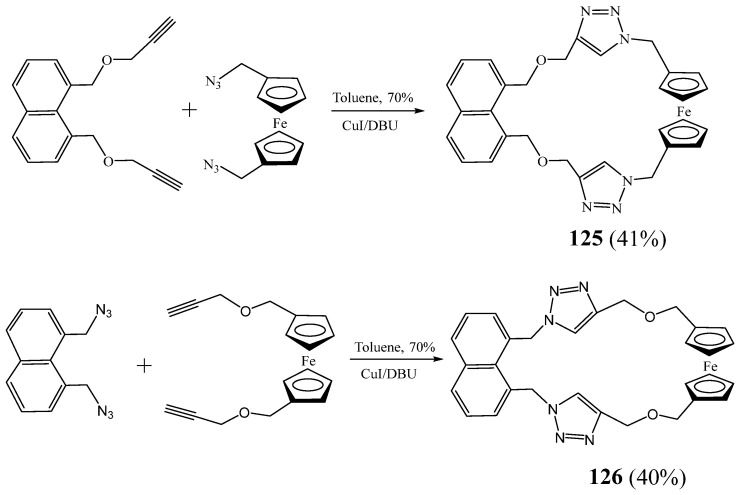
Ferrocene-containing macrocyclic triazoles **125** and **126** (Reprinted with permission from ref. [52]. Copyright 2016 Elsevier).

Li et al. [52] described the synthesis of two novel ferrocene-containing macrocyclic triazoles **125** and **126** using a ‘click’ reaction (Figure 31). The anions binding abilities of these macrocycles were evaluated, and results revealed that these receptors have exclusive electrochemical sensing of H_2_PO_4_^−^.

**Figure 32:**
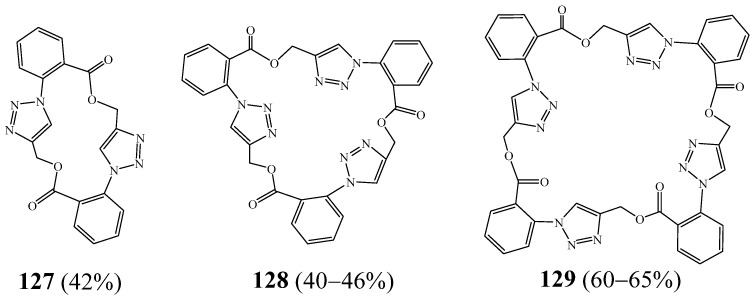
Macrocycles containing two, three, and four 1,2,3-triazole motifs **127**–**129** (Reprinted with permission from ref. [53]. Copyright 2012 © Georg Thieme Verlag KG).

Hradilová et al. [53] developed a new approach for the preparation of macrocycles containing two, three, and four 1,2,3-triazole motifs **127**–**129** from simple compounds such as 2-azidobenzoic acid, propargyl bromide, and propargyl anthranilate (Figure 32). The macrocyclic precursor was constructed by a series of steps which include cycloaddition of an azide with an alkyne, alkylation of a carboxylic acid with propargyl bromide, and formation of an azide from an amino group.

**Figure 33:**
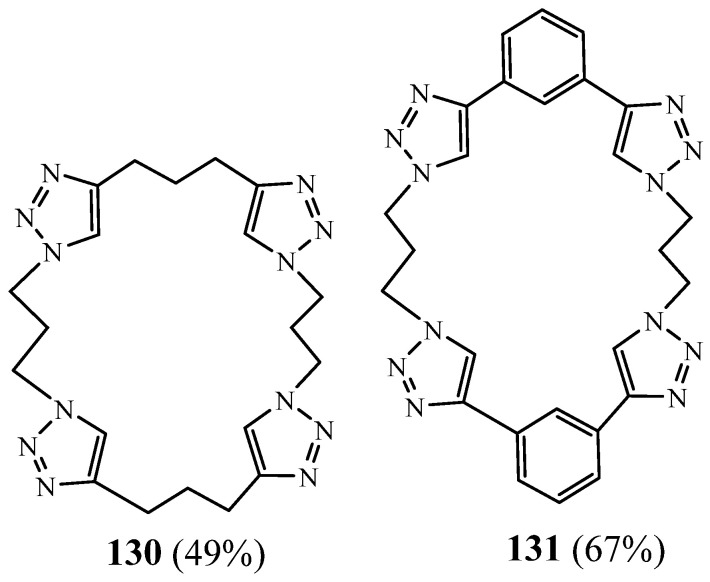
Triazolic macrocycles **130** and **131** with good anion-complexing properties (Reprinted with permission from ref. [54]. Copyright 2012 Royal Society of Chemistry).

White et al. [54] conveyed the synthesis of two tetra-1,2,3 triazole macrocycles **130** and **131** in good yields using the copper(I)-catalysed cycloaddition of bis-triazole azides and bis-alkynes (Figure 33). One of them was alkylated to give a cyclic tetra-triazolium receptor, which complexed anions strongly in competitive DMSO–water mixtures. In 1:1 DMSO–water, the tetracationic receptor exhibited a preference for the larger halides, bromide, and iodide.

**Figure 34:**
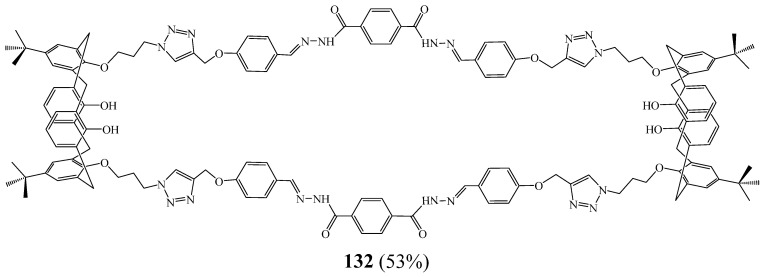
Structure of macrocycle **132** as selective sensor towards Cu^2+^ (Reprinted with permission from ref. [55]. Copyright 2016 Elsevier).

Khan et al. [55] presented the elaboration of a novel fluorescent bis-calix[4]arene macrocycle **132** bearing four 1,2,3 triazole rings and incorporating metal-binding pockets (Figure 34). The structures of this macrocycle and its precursors were checked via NMR and MS, as well as X-ray crystallography. Macrocycle **132** displayed selective fluorescence quenching after interacting with Cu^2+^ in the presence competing metal cations including Mg^2+^, Ca^2+^, Ba^2+^, Ag^+^, Zn^2+^, Ti^4+^,Cd^2+^, Hg^2+^, Pb^2+^, In^3+^, La^3+^, Cr^3+^, Ni^2+^, Sb^3+^, V^5+^, Fe^3+^, Co^2+^, Sn^2+^, Sn^2+^, and Tl^+^. The Cu^2+^ limit of detection was found to be 40 Nm, much lower than its threshold level (∼20 μM) in drinking water permitted by the US Environmental Protection Agency (EPA). Furthermore, drinking water samples from Karachi University (Pakistan), spiked with Cu^2+^, were analysed with the sensing system, and the results showed an excellent agreement with the fluorescence quenching phenomenon of the macrocycle examined in deionised water. Importantly, it could be used to detect Cu^2+^ in living cells.

## 6. Synthesis of Pyrazolic Macrocycles

**Figure 35:**
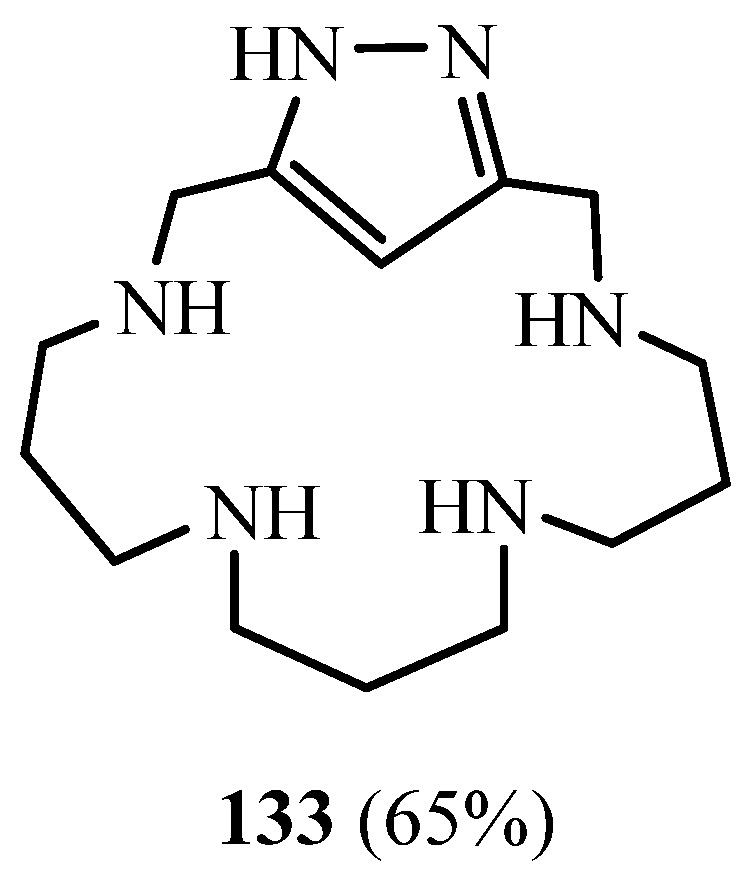
The structure of 1*H*-pyrazole cyclophane **133** (Reprinted with permission from ref. [56]. Copyright 2013 American Chemical Societ).

Belda et al. [56] reported the synthesis of a novel cyclophane **133** consisting of a 1*H*-pyrazole moiety linked through methylene groups to a 1,5,9,13-tetraazadecane chain (Figure 35). According to Belda et al., this is one of the first reported syntheses of a [1+1] condensation 1*H*-pyrazole azamacrocyclic ligand. This macrocycle was obtained by a macrocyclisation reaction of the tosylated polyamine with either 1*H*-3,5-bis(chloromethyl)pyrazole in CH_3_CN using K_2_CO_3_ as a base. The crystal structures of the corresponding copper II complexes show that Cu^2+^ coordination leads to the formation of 2:2 Cu^2+^:L dinuclear dimeric complexes in which the 1*H*-pyrazole units lose a proton behaving as bis(monodentate) bridging ligands.

Ashok et al. [57] conveyed an efficient approach to the synthesis of fused pyrazole-annulated macrocycles. This was performed by Vilsmeier–Haack reaction of substituted *o*-hydroxyacetophenones with phenylhydrazine, followed by reduction of the resulting pyrazolyl aldehydes yielded the corresponding alcohols. These precursors upon alkylation with dibromoalkanes gave the target library. the final macrocycles were screened for their antimicrobial activity. This investigation revealed that most of the tested compounds displayed some inhibitory effects on the growth of the tested Gram-positive and Gram-negative bacterial strains, while a low inhibitory activity against the tested fungal strains was observed.

**Figure 36:**
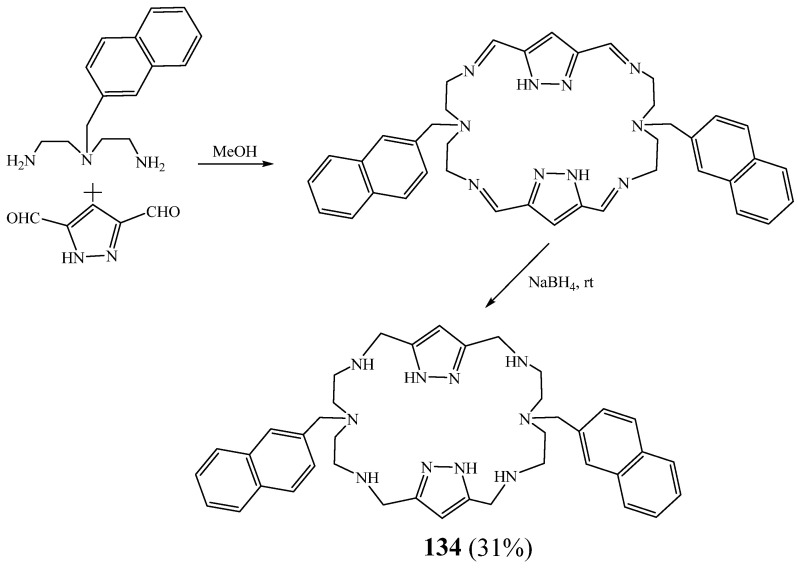
Synthetic pathway of bipyrazolic macrocycle **134** (Reprinted with permission from ref. [58]. Copyright 2010 Royal Society of Chemistry).

Javier Pitarch et al. [58] described the synthesis of a new macrocycle **134** obtained by dipodal [2+2] condensation of the polyamine 3-(naphthalen-2-ylmethyl)pentane-1,5-diamine with 1*H*-pyrazole-3,5-dicarbaldehyde, followed by a reduction using NaBH_4_ (Figure 36). This macrocycle presented five measurable protonation steps in the 2.0–11.0 pH range. Through fluorescence emission studies, they found that the Zn^2+^ coordination promotes a boat-like shape conformation that approaches both fluorophores and facilitates the formation of an excimer which reaches its highest emission for a 1:1 (Zn^2+^:**159**) molar ratio.

**Figure 37:**
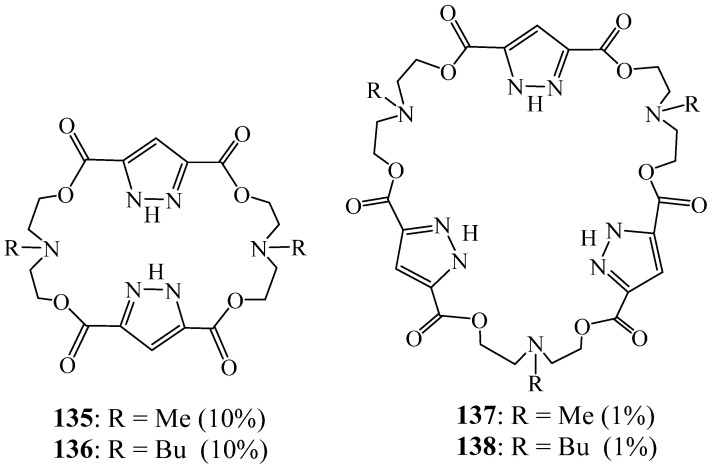
Macrocycles **135**–**138** containing two and three pyrazolic moieties (Reprinted with permission from ref. [59]. Copyright 2011 American Chemical Societ).

Reviriego et al. [59] used an improved synthetic method to synthesise 26-membered diaza tetraester crowns (**135**, **136**) and 39-membered triaza hexaester crowns (**137**, **138**) containing two and three pyrazolic moieties, respectively (Figure 37). This was performed by reacting the cyclic stannoxanes obtained from RN-diethanolamine (R = Me, Bu) and dibutyltin oxide 1*H*-pyrazole-3,5-dicarbonyl dichloride. The new structures were confirmed by their analytical and spectroscopic data. Both diaza tetraester crowns **135** and **136**, containing two 1*H*-pyrazole units, self-assembled into dimeric species through the formation of four hydrogen bonds involving the two NH pyrazole groups and the two tertiary amine groups of both crowns, as proved by X-ray crystallography and NMR analysis.

**Figure 38:**
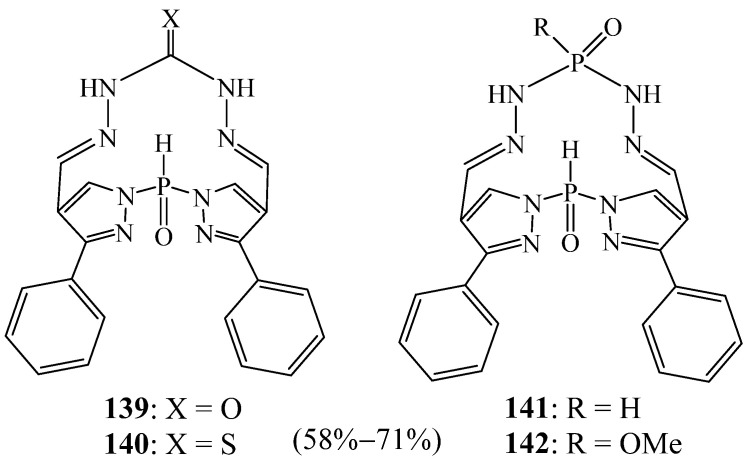
Phosphorus bipyrazolic macrocycles **139**–**142** (Reprinted with permission from ref. [60]. Copyright 2013 TÜBİTAK).

Ali et al. [60] reported a simple synthetic method for the preparation of four new phosphorus macrocycles **139**–**142** (Figure 38) in which the pyrazole rings are appended to a phosphorus atom. The methodology was based on the cyclocondensation reaction of *bis*(4-formylpyrazolyl) phosphine oxides with nitrogen nucleophiles that contain active terminal amino groups. A preliminary antimicrobial evaluation of the tested compounds showed that they had low-to-moderate activities, compared with the reference drugs.

**Figure 39:**
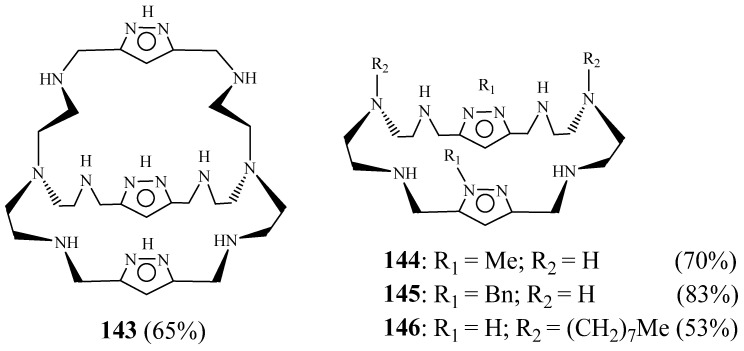
Structure of macrocycles **143**–**146** (Reprinted with permission from ref. [61]. Copyright 2012 American Chemical Societ).

Sanchez-Moreno et al. [61] elaborated pyrazole-containing macrobicyclic polyamine **143** and three pyrazole-containing monocyclic polyamines **144**–**146** (Figure 39). Bicyclic macrocycle **168** and monocyclic polyamine containing two pyrazole units were obtained principally via the condensation of 1*H*-pyrazole-3,5-dicarbaldehyde with tris(2-aminoethyl)amine and Bis(2-aminoethyl)amine, respectively, followed by further reaction steps. The in vitro and in vivo anti-*Trypanosoma cruzi* activity was studied. The compounds were more active against the parasite and less toxic against Vero cells than the reference drug benznidazole; in addition, **144** and **145** were especially effective, whereas cryptand **144** was the most active, particularly in the chronic phase.

These compounds were also assayed on *Leishmania infantum* and *Leishmania braziliensis* species [62]. There were found more active and less toxic than glucantime. Both infection rates and ultrastructural alterations confirmed that **143** and **145** were highly leishmanicidal and induced extensive parasite cell damage. Modifications in the excretion products of parasites treated with **143**–**145** were also consistent with substantial cytoplasm alterations. Compound **145** was highlighted as a potent inhibitor of Fe-SOD in both species, whereas its effect on human CuZn-SOD was poor.

In 1997, Bol et al. [63] established the synthesis of two new tetrapyrazolic macrocycles with two pyridyl lateral arms by condensation of 1,n bis(3′-chloromethyl-5′-methyl-l′-pyrazolyl) alkane (*n* = 2 or 3) with the 1,n bis(3′pyridyl-2-ylethylamino)-5′-methyl-l′-pyrazolyl) alkane (*n* = 2 or 3) in tetrahydrofuran and acetonitrile, respectively. These two macrocycles formed stable copper I, copper II and Zinc II complexes.

**Figure 40:**
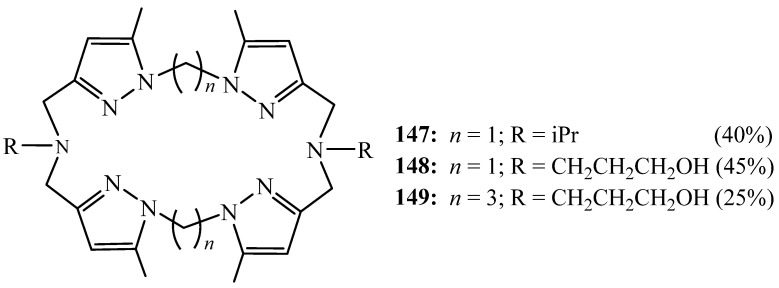
Tetrapyrazolic macrocycles **147**–**149** with functionalised lateral arms.

Malek et al. [64] reported the synthesis of another family of new symmetrical tetrapyrazolic macrocycles **147**–**149**, also bearing two lateral arms. They were obtained through a [2+2] cyclocondensation of a primary amine with the 1,n bis(3′-chloromethyl-5′-methyl-l′-pyrazolyl) alkane (*n* = 1, 3) in acetonitrile using the high dilution condition (Figure 40). In the case of *n* = 3, they also observed the formation of the macrocycle resulting from [1+1] cyclisation due to the flexibility of the chlorinated derivative.

The macrocycle with isopropyl lateral arms can also be obtained by a 2 + 2 reaction of a tripodal ligand and dibromomethane using phase transfer catalysis (PTC) in high-dilution conditions. Nevertheless, the yield of the desired macrocycle remained practically unchangeable. These tetrapyrazolic macrocycles were found to be able to complex and transport across a solid membrane selectively the K^+^ cation.

Cherfi et al. [65] also conveyed the synthesis of another macrocycle with two long flexible lateral arms bearing a donor group using the same method reported by Malek et al. [64]. They found that the ability of this macrocycle to extra selectively K^+^ is improved with the increasing the lateral arm length.

**Figure 41:**
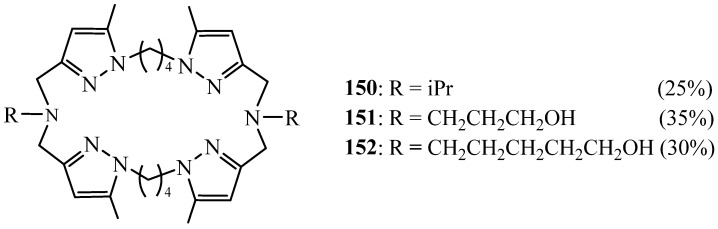
Other tetrapyrazolic macrocycles **150**–**152** with two sidearms.

As a continuation of these studies, Harit et al. [66,67] synthesised a new generation of bi-functionalised tetrapyrazolic macrocycles **150**–**152** by reaction of 1,4-bis(3′-chloromethyl-5′-methyl-l′-pyrazolyl) butane with primary amines with the aim to increase the cavity size of these compounds (Figure 41). Indeed, the resulting compound was found to be complex and also transport selectively the Cs^+^ cation.

They also elaborated a new generation of this kind of macrocycles using the same method and changing the bis-chlorinated derivative (*n* = 1) by its brominated homologue to improve the cyclisation reaction yield. However, no particular change was obtained [68]. Besides their ability to complex selectively K^+^, they possessed some antibacterial activity (32 μg/mL) against both Gram-positive and Gram-negative bacteria. Other homologues of macrocycles reported by Bol et al. [63] were also synthesised. They also showed an affinity to complex K^+^ cation and some antibacterial activity [69].

**Figure 42:**
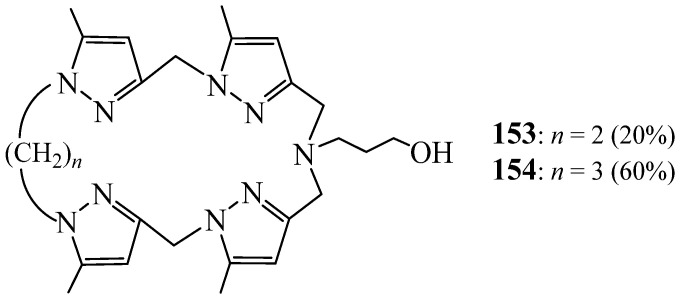
Tetrapyrazolic macrocycles **153** and **154** with one lateral arm (Reprinted with permission from ref. [71] Copyright 2006 Elsevier; Reprinted with permission from ref. [71] Copyright 2004 Elsevier).

Radi et al. [70,71] used the same strategy performed by Malek et al. [64] to synthesise two tetrapyrazolic macrocycles **153** and **154** with different cavity sizes and bear one lateral arm (Figure 42). This was performed by [1+1] cyclisation of a chlorinated derivative with 3 aminopropan-1-ol to obtain the macrocycle **154**, and the 1,2 dibromoethane with a tetrapod ligand to obtain the macrocycle **153**. These macrocycles were also found to have a complexing affinity towards both alkali and heavy metal cations.

**Figure 43:**
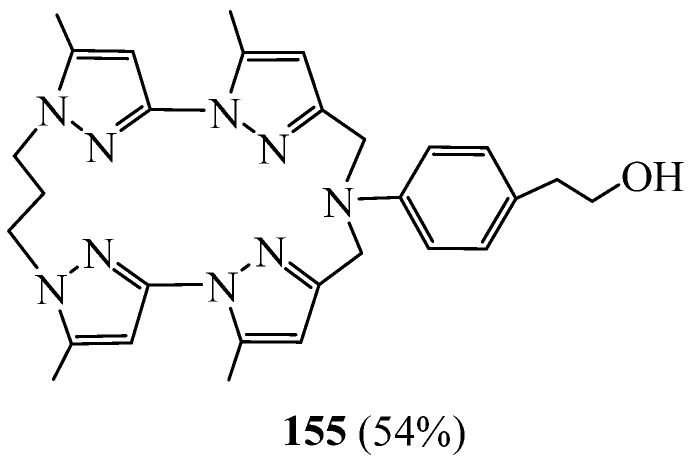
Tetrapyrazolic macrocycles **155** with one aromatic lateral arm.

As a continuation of the studies of Tarrago [72], Harit et al. [73] elaborated a new macrocycle **155** with an aromatic lateral arm bearing a hydroxyl group (Figure 43). The synthesis was achieved via two pathways and led to two different yields. The study of the complexing properties of this macrocycle towards the alkali metal ions (Li^+^, Na^+^, K^+^, Cs^+^) showed remarkable extraction and transport [74] selectivities for the lithium cation in competitive conditions.

**Figure 44:**
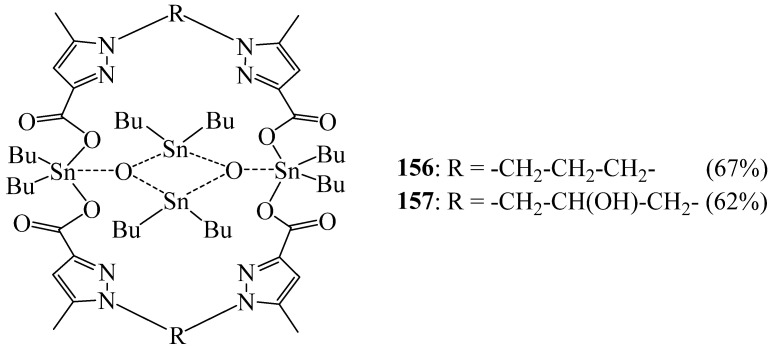
Organotin (IV) bipyrazole-dicarboxylate macrocyclic complexes **156** and **157**.

Recently, Dahmani et al. [75] reported two new organotin (IV) bipyrazole-dicarboxylate macrocyclic complexes **156** and **157** (Figure 44) by condensing of one equivalent of the bipyrazole-dicarboxylic acids 1,1′-(propane-1,3-diyl)bis(5-methyl-1*H*-pyrazole-3-carboxylic acid) or 1,1′-(2-hydroxypropane-1,3-diyl)bis(5-methyl-1*H*-pyrazole-3-carboxylic acid) with two equivalents of oxide di-(n-butyl)tin. These macrocycles possess an interesting fungicidal activity against the pathogenic strain *Fusarium oxysporum f.* sp. *albedinis*.

## 7. Conclusions

In summary, the aim of this review was to give readers an overview of some methods for the synthesis of several N-heterocyclic five-membered ring structures (Imidazole, triazole, tetrazole, or pyrazole) containing macrocycles, as well as their applications in different fields such as pharmacology, biology, and complexation of alkali or transition metal cations. The reaction conditions considerably affect the size and conformation, granting access to a new range of macrocyclic architectures. It was revealed that the synthesis of macrocyclic molecules is a promising area that continues to grow year by year, giving the opportunity to design new active agents with excellent biological and complexing properties.

## Data Availability

Not applicable.
